# Association between plasminogen activator inhibitor-1 (*PAI-1*) 4G/5G polymorphism and risk of Alzheimer's disease, metabolic syndrome, and female infertility: A meta-analysis

**DOI:** 10.1097/MD.0000000000023660

**Published:** 2020-12-11

**Authors:** Xin Zhang, Bai Gao, Bing Xu

**Affiliations:** a Department of Neurology, Shenyang First People's Hospital, Dadong District; b Department of Nerve Function, ShengJing Hospital of China Medical University, Heping District, Shenyang, Liaoning Province, People's Republic of China.

**Keywords:** Alzheimer's disease, female infertility, meta-analysis, metabolic syndrome, plasminogen activator inhibitor-1, polymorphism

## Abstract

**Background:**

Plasminogen activator inhibitor-1 (PAI-1) is considered to be involved in the physiopathological mechanisms of Alzheimer's disease (AD), metabolic syndrome (MetS), and female infertility. Previous studies investigating the association between *PAI-1*4G/5G (rs1799889) gene polymorphism and the risk of AD, MetS, and female infertility have reported inconsistent results. The aim of the present study was to investigate possible associations.

**Methods:**

Eligible studies were retrieved through PubMed, Medline, EMBASE, CNKI, and WANFANG databases. The odds ratios (ORs) and 95% confidence intervals (CIs) were used to assess the associations. Subgroup analyses by ethnicity and mean age, sensitivity analyses, and publication bias were performed.

**Results:**

Five studies (four articles) for AD, six studies (six articles) for MetS, and four studies (four articles) for female infertility were included in this meta-analysis. Our results showed no significant associations between the *PAI-1*4G/5G polymorphism and the risk of AD and female infertility in five genetic models. For the risk of MetS, the *PAI-1* 4G/5G (rs1799889) polymorphism may be associated with the risk of MetS (4G vs 5G, OR = 1.31, 95%CI = 1.04–1.64, *P* = .021), especially in Asians (4G/4G vs 4G/5G+5G/5G, OR = 1.38, 95%CI = 1.01–1.87, *P* = .041) and patients with mean age > 50 years old (4G/4G vs 4G/5G+5G/5G, OR = 1.36, 95%CI = 1.03–1.78, *P* = .029).

**Conclusion:**

The present meta-analysis suggested that the *PAI-1* 4G/5G polymorphism might be associated with the risk of MetS, but no evidence was detected for AD and female infertility.

## 1 Introduction

Alzheimer's disease (AD) is a complicated neurodegenerative disorder characterized by vascular amyloid-β (Aβ) deposition, neurofibrillary tangles, and nerve cell death, particularly in some areas of the cerebrum memory and more advanced administrative performance.^[1,2]^ Cognitive dysfunction and memory impairment are the main signs and symptoms of AD.^[3,4]^ Metabolic syndrome (MetS) is characterized by a cluster of multiple metabolic abnormalities, including central obesity, hypertension, dyslipidemia, and elevated glucose.^[5]^ Infertility is defined as the failure to conceive after 1 year of regular sexual intercourse, affecting ∼10% to 20% of couples.^[6,7]^ Of all infertility cases, ∼50% are associated with female factors.^[8]^ Besides environmental risk factors, genetic variation may play a key role in the pathogenesis of AD, MetS, and female infertility.

Plasminogen activator inhibitor type-1 (PAI-1), also known as protein serpin peptidase inhibitor, clade E, member 1 (SERPINE1), belongs to the serine protease inhibitor superfamily.^[9]^ It plays a critical role in the regulation of the fibrinolytic system by rapidly inhibiting the tissue-type plasminogen activator (tPA) and urokinase-type plasminogen activator (uPA), which cannot cleave plasminogen to give plasmin.^[10]^ The gene coding for PAI-1 has several polymorphic loci, among which the most studied is the 4G/5G insertion/deletion polymorphism containing either 4 or 5(4G/5G) guanine bases at −675 of the *PAI-1* promoter.^[11,12]^ When *PAI-1* gene mutations occur, PAI-1 levels will be increased, leading to reduced plasma fibrinolytic activity, which has been observed in a variety of diseases, including AD, MetS, infertility, etc.

Up to date, the associations between the *PAI-1* 4G/5G polymorphism and the risk of AD, MetS, and female infertility have been investigated by many studies, whereas the results were still inconsistent due to various genetic backgrounds, small sample sizes of each study, and possible biases. To increase statistical power and identify the association between the *PAI-1* 4G/5G polymorphism and the risk of AD, MetS, and female infertility, a meta-analysis was performed.

## 2 Methods

This meta-analysis was conducted and reported according to the Preferred Reporting Items for Systematic Reviews and Meta-Analyses (PRISMA) 2009 checklist.^[13]^

### 2.1 Literature search strategy

We searched PubMed, Medline, Embase, CNKI, and WANFANG databases and references of relevant publications only in English and Chinese up to October 2019. Using both medical subject heading terms (“Alzheimer Disease,” “Metabolic Syndrome,” “infertility,” “polymorphism, genetic,” “Plasminogen Activator Inhibitor 1”) and text words (“Alzheimer Disease,” “Alzheimer's Disease,” “metabolic syndrome,” “infertility,” “polymorphism,” “genetic,” “genetic polymorphism,” “polymorphism,” “Plasminogen Activator Inhibitor-1,” “PAI-1,” “Serpin E1,” “SERPINE1 Protein,” “Type 1 Plasminogen Activator Inhibitor”). Our study was approved by the Medical Ethics Committee of Shenyang First People's Hospital.

### 2.2 Inclusion and exclusion criteria

Eligible articles had to meet the following criteria:

1. articles about *PAI-1* gene polymorphisms and risk of AD, MetS, and infertility;
2. sufficient genotype frequencies for estimating odds ratios (ORs) with 95% confidence intervals (CIs); and
3. case–control study.

Exclusion criteria included:

1. insufficient genotype data;
2. duplicate studies;
3. case reports, reviews, editorials, comments, and abstracts.

### 2.3 Data extraction and study quality assessment

The following data were extracted from each study: first author, year of publication, mean age, country, ethnicity, genotyping method, number of cases and controls, genotype frequencies in cases and controls, the *P*-value of Hardy-Weinberg equilibrium (HWE) test in controls, and Newcastle-Ottawa Scale (NOS) score.^[14]^ Two authors independently extracted data and evaluated the study quality using NOS. Total NOS scores ranged from zero to nine stars. A NOS score of 6 to 9 is considered to be of high methodological quality. Discrepancies were resolved through discussion.

### 2.4 Statistical analysis

In the present meta-analysis, the risks of the associations between *PAI-1* 4G/5G genetic variants and AD, MetS, and female infertility were reported as ORs with 95% CIs. The ORs and 95%CIs of risks were estimated in allelic, dominant, recessive, heterozygote, and homozygote models for each study. Statistical heterogeneity was quantified using Chi-square-based *Q* statistics and *I*^2^ values. If *P* > .10 and *I*^2^ < 50% indicated significant heterogeneity, fixed-effects models (Mantel-Haenszel method) were used^[15]^; otherwise, the random-effects models (Mantel-Haenszel method) were merged.^[16]^ The significance of the *overall OR was estimated by the Z* test. Stratified analyses based on ethnicity and mean age were conducted. HWE was tested in control groups using the Chi-square test. Egger's linear regression test was performed to test for publication bias.^[17]^ If there was publication bias, we recalculated the adjusted ORs using the trim-and-fill method.^[18]^ to evaluate the possible impact of publication bias. Sensitivity analyses were performed by sequentially omitting each study to evaluate the stability and reliability of the results. All statistical analyses were conducted using Stata software (Version 15.0, Stata Corp, College Station, TX). Value of *P* < .05 was considered as significant difference.

## 3 Results

### 3.1 Study selection

**Figure 1:**
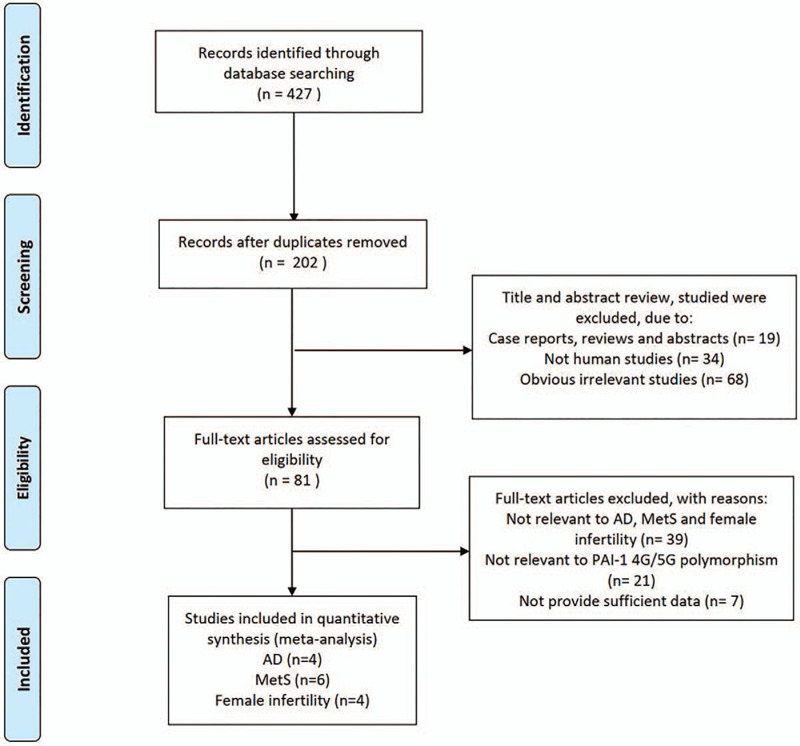
Flow chart of the systematic search process.

Our research yielded 427 studies of potentially relevant studies. After screening, 15 studies (14 articles) were finally enrolled in this meta-analysis.^[19–32]^ The study screening process is shown in Figure 1. Of these 14 articles, four articles (five studies) for AD, six articles (six studies) for MetS, and four articles (four studies) for female infertility were included in this meta-analysis. The corresponding characteristics and *P-*values for the HWE and Newcastle-Ottawa scale scores from all included studies are listed in Table 1.

**Table 1:** Summary of included studies for *PAI-1*(rs1799889) polymorphism.

						Cases			Control					
Author	Year	Age	Country	Ethnicity	Sample size (case/control)	4G/4G	4G/5G	5G/5G	4G/4G	4G/5G	5G/5G	Genotyping method	HWE *P*	NOS score
Alzheimer's disease														
Fekih-Mrissa N	2017	75.2 ± 5.3	Tunisia	Asian	60/120	17	33	10	12	46	62	PCR	.429	7
Lu Y	2009	71.2 ± 9.4	China	Asian	324/278	91	166	67	89	143	46	PCR	.368	8
Shibata N	2007	70.0 ± 9.0	North Europe	Caucasian	192/195	50	90	52	31	58	96	NA	<.001	6
Shibata N	2007	NA	Canada	Caucasian	189/245	52	86	51	64	131	50	NA	.254	6
Clarimón J	2003	76.6 ± 5.3	Spain	Hispanic	136/87	29	78	29	21	48	18	PCR	.329	8
Metabolic Syndrome														
Zhang LQ	2018	25.1 ± 5.2	China	Asian	15/15	6	6	3	2	7	6	NA	.985	6
Aburto-Mejía E	2017	56.0 ± 10.0	Mexico	Mestizo	215/307	25	104	86	26	159	122	PCR	.009	6
Sun SK	2013	59.0 ± 7.9	China	Asian	272/92	88	106	78	20	36	36	PCR	.064	6
Al-Hamodi Z	2012	51.9 ± 6.9	Yemen	Asian	227/131	57	109	61	30	63	38	PCR	.692	5
Bouchard L	2010	35.0 ± 7.0	Canada	Caucasian	50/39	16	30	4	8	16	15	PCR	.342	3
Zhao SH	2005	54.0 ± 12.0	China	Asian	160/90	56	76	28	27	47	16	PCR	.567	6
Female infertility														
Djurovic J	2017	35.9 ± 6.8	Serbia	Caucasian	1080/133	325	546	209	46	67	20	PCR	.583	6
Kydonopoulou K	2017	25.0–48.0	Greece	Caucasian	115/107	33	56	26	21	44	42	PCR	.135	7
Gonçalves-Filho RP	2011	34.4 ± 4.1	Brazil	Caucasian	64/148	19	22	23	36	50	62	PCR	<.001	5
Sun JH	2008	26.0 ± 9.0	China	Asian	105/85	51	37	17	19	42	24	PCR	.939	5

In each test, a *P*<0.05 was considered statistically significant. HWE = Hardy-Weinberg equilibrium, NA = not available, NOS = Newcastle–Ottawa scale, PAI-1 = Plasminogen Activator Inhibitor 1, PCR = polymerase chain reaction.

### 3.2 Association of the *PAI-1* 4G/5G polymorphism with AD

To determine the potential association between the *PAI-1* 4G/5G polymorphism and the risk of AD, 5 studies (4 articles) including 901 patients and 925 controls were enrolled in this meta-analysis. As shown in Table 2, this result suggested that the *PAI-1* 4G/5G polymorphism was not associated with the risk of AD in five genetic models. Furthermore, stratification by ethnicity failed to explore any association between the polymorphism and AD in Asians and Caucasians.

**Table 2:** Main results of the meta-analysis.

			Test of heterogeneity		Test of association			
Categories	Study number	Comparison model	*P*	*I*^2^ (%)	OR (95% CI)	*P*	Egger's test (*P*)	Effects model
Alzheimer's Disease								
Overall	5	4G vs 5G	<.001	90.8	1.32 (0.84, 2.09)	.225	.241	Random
	5	4G/4G vs 5G/5G	<.001	87.9	1.57 (0.70, 3.54)	.277	.295	Random
	5	4G/5G vs 5G/5G	<.001	88.0	1.42 (0.70, 2.87)	.335	.464	Random
	5	4G/4G vs 4G/5G+5G/5G	.007	71.9	1.27 (0.82, 1.96)	.278	.175	Random
	5	4G/4G+4G/5G vs 5G/5G	<.001	90.3	1.47 (0.70, 3.08)	.305	.515	Random
Asian	2	4G vs 5G	<.001	95.9	1.59 (0.45, 5.60)	.471	NA	Random
	2	4G/4G vs 5G/5G	<.001	95.0	2.39 (0.20, 28.38)	.491	NA	Random
	2	4G/5G vs 5G/5G	<.001	92.7	1.82 (0.34, 9.82)	.487	NA	Random
	2	4G/4G vs 4G/5G+5G/5G	.001	90.3	1.64 (0.39, 6.79)	.498	NA	Random
	2	4G/4G+4G/5G vs 5G/5G	<.001	94.8	1.96 (0.29, 13.34)	.491	NA	Random
Caucasian	2	4G vs 5G	<.001	93.8	1.35 (0.61, 3.01)	.459	NA	Random
	2	4G/4G vs 5G/5G	.001	91.0	1.54 (0.42, 5.59)	.515	NA	Random
	2	4G/5G vs 5G/5G	<.001	94.8	1.36 (0.31, 5.87)	.682	NA	Random
	2	4G/4G vs 4G/5G+5G/5G	.147	52.4	1.35 (0.83, 2.17)	.223	NA	Random
	2	4G/4G+4G/5G vs 5G/5G	<.001	95.1	1.42 (0.35, 5.78)	.623	NA	Random
Metabolic Syndrome								
Overall	6	4G vs 5G	.076	49.9	1.31 (1.04, 1.64)	.021	.049	Random
	6	4G/4G vs 5G/5G	.126	41.9	1.71 (1.10, 2.65)	.016	.071	Random
	6	4G/5G vs 5G/5G	.070	50.9	1.25 (0.84, 1.84)	.268	.124	Random
	6	4G/4G vs 4G/5G+5G/5G	.678	0.0	1.41 (1.09, 1.83)	.009	.053	Random
	6	4G/4G+4G/5G vs 5G/5G	.034	58.6	1.40 (0.94, 2.09)	.098	.087	Random
Asian	4	4G vs 5G	.222	31.6	1.27 (1.00, 1.63)	.050	.323	Fixed
	4	4G/4G vs 5G/5G	.319	14.5	1.52 (1.00, 2.29)	.049	.376	Fixed
	4	4G/5G vs 5G/5G	.807	0.0	1.15 (0.83, 1.59)	.396	.739	Fixed
	4	4G/4G vs 4G/5G+5G/5G	.414	0.0	1.38 (1.01, 1.87)	.041	.205	Fixed
	4	4G/4G+4G/5G vs 5G/5G	.509	0.0	1.29 (0.95, 1.74)	.099	.589	Fixed
Age > 50 years	4	4G vs 5G	.364	5.7	1.17 (1.00, 1.36)	.051	.507	Fixed
	4	4G/4G vs 5G/5G	.606	0.0	1.42 (1.03, 1.96)	.031	.775	Fixed
	4	4G/5G vs 5G/5G	.704	0.0	1.04 (0.81, 1.33)	.768	.640	Fixed
	4	4G/4G vs 4G/5G+5G/5G	.727	0.0	1.36 (1.03, 1.78)	.029	.334	Fixed
	4	4G/4G+4G/5G vs 5G/5G	.471	0.0	1.13 (0.90, 1.43)	.296	.696	Fixed
Female infertility								
Overall	4	4G vs 5G	<.001	84.0	1.38 (0.88, 2.17)	.159	.120	Random
	4	4G/4G vs 5G/5G	.002	79.9	1.69 (0.77, 3.68)	.189	.046	Random
	4	4G/5G vs 5G/5G	.144	44.6	1.21 (0.79, 1.87)	.383	.491	Random
	4	4G/4G vs 4G/5G+5G/5G	.002	79.2	1.51 (0.81, 2.79)	.193	.172	Random
	4	4G/4G+4G/5G vs 5G/5G	.021	69.1	1.40 (0.82, 2.39)	.214	.215	Random
Caucasian	3	4G vs 5G	.008	79.2	1.19 (0.77, 1.84)	.443	.340	Random
	3	4G/4G vs 5G/5G	.015	76.0	1.31 (0.59, 2.88)	.506	.246	Random
	3	4G/5G vs 5G/5G	.067	62.9	1.22 (0.68, 2.17)	.510	.552	Random
	3	4G/4G vs 4G/5G+5G/5G	.125	52.0	1.14 (0.72, 1.79)	.580	.202	Random
	3	4G/4G+4G/5G vs 5G/5G	.019	74.7	1.26 (0.66, 2.41)	.480	.407	Random

CI = confidence interval, OR = odds ratio.

### 3.3 Association of the *PAI-1* 4G/5G polymorphism with MetS

To determine the potential association between the *PAI-1* 4G/5G polymorphism and the risk of MetS, 6 studies including 939 patients and 674 controls were enrolled in this meta-analysis. We found a significant association between the *PAI-1* 4G/5G polymorphism and MetS risk in the overall population under allele (4G vs 5G, OR = 1.31, 95% CI = 1.04–1.64, *P* = .021), homozygous (4G/4G vs 5G/5G, OR = 1.71, 95% CI = 1.10–2.65, *P* = .016), and dominant model (4G/4G vs 4G/5G+5G/5G, OR = 1.41, 95% CI = 1.09–1.83, *P* = .009). Furthermore, stratification by ethnicity identified a significant association between this polymorphism and MetS in Asians under homozygous (4G/4G vs 5G/5G, OR = 1.52, 95% CI = 1.00–2.29, *P* = .049) and dominant model (4G/4G vs 4G/5G+5G/5G, OR = 1.38, 95% CI = 1.01–1.87, *P* = .041) (Table 2). When stratified by mean age, a significantly increased risk of MetS was found in patients older than 50 years (4G/4G vs 4G/5G+5G/5G, OR = 1.36, 95%CI = 1.03–1.78, *P* = .029).

### 3.4 Association of the *PAI-1* 4G/5G polymorphism with female infertility

To determine the potential association between the *PAI-1* 4G/5G polymorphism and the risk of female infertility, 4 studies including 1364 patients and 473 controls were enrolled in this meta-analysis. As shown in Table 2, no association was found between the *PAI-1* 4G/5G polymorphism and female infertility risk in the overall population under any genetic model. Furthermore, stratification by ethnicity failed to explore any association between the polymorphism and infertility in Caucasians.

### 3.5 Publication bias, sensitivity analyses

Egger's test method was used to assess publication bias. No evident publication bias was detected among the studies on the association of the *PAI-1* 4G/5G polymorphism and AD risk (Table 2). However, publication bias was detected for the *PAI-1* 4G/5G polymorphism, MetS, and infertility. After adjustment with the trim and fill method, the adjusted pooled OR was changed (4G vs 5G, OR = 1.18, 95% CI = 0.91–1.52) compared with unadjusted pooled OR (OR = 1.31, 95%CI = 1.04–1.64), but this was not significant, indicating that the results were not robust. For female infertility, the trim and fill method revealed no difference between the adjusted pooled OR and 95% CI (4G/4G vs 5G/5G, OR = 1.69, 95% CI = 0.77–3.68) and the unadjusted results (4G/4G vs 5G/5G, OR = 1.69, 95% CI = 0.77–3.68), which indicated that the presence of publication bias did not influence the stability of the results. Sensitivity analyses showed that several studies had significant impact on corresponding pooled effects, which did not confirm the stability of our meta-analysis (data not shown).

## 4 Discussion

We performed a meta-analysis to investigate the association between the *PAI-1* 4G/5G polymorphism and the risk of AD, MetS, and female infertility. The present meta-analysis suggested that the *PAI-1* 4G/5G polymorphism might be associated with the risk of MetS, but no evidence was detected for AD and female infertility. Besides the overall analysis, subgroup analyses were also performed according to ethnicity and age. A significantly increased risk of MetS was found in Asians and patients with a mean age > 50 years.

Amyloid plaque deposits composed primarily of Aβ peptides play an important role in the pathology of AD.^[33]^ Plasmin, one of the proteases, is known for its role in regulating Aβ levels.^[34]^ Plasmin formation is mediated by plasminogen activators (tPA and uPA) that cleave the plasminogen to the active plasmin. PAI-1 expression inhibits both tPA and uPA and their activity, thus decreasing the level of plasmin.^[35]^ The 4G/5G polymorphism, which is located at the transcription site of the *PAI-1* gene, is responsible for increased PAI-1 expression.^[11]^ Thus, the *PAI-1* 4G/5G polymorphism may be associated with the pathogenesis of AD. It is well known that increased plasma PAI-1 expression and decreased tPA activity are thought to play important roles in the development of MetS.^[36]^ Increased PAI-1 levels have been shown to be associated with many risk factors such as central obesity, hypertension, dyslipidemia, and glucose intolerance, collectively referred to as MetS.^[27,37–39]^

Several limitations of the present meta-analysis should be considered. First, our study included five studies on AD, six studies for MetS, and four studies for female infertility. The meta-analysis may be unable to have sufficient power to identify real associations because of the limited study number, especially when grouped by ethnicity and age. Second, we observed the heterogeneity of the *PAI-1* 4G/5G polymorphism in the overall population and subpopulations of AD and female infertility, and could not identify the sources of heterogeneity by stratified analysis based on ethnicity. Third, selection bias may occur due to the inclusion of only English or Chinese literature. Fourth, AD, MetS, and female infertility are multifactorial disorders resulting from complex interactions between genetic, epigenetic, and environmental factors, suggesting that the *PAI-1* polymorphism may only partially play a role in the pathogenesis of these diseases, which may result in bias in our results. Finally, our study only determined the associations between a single locus in the *PAI-1* gene 4G/5G polymorphisms and three diseases in patients, but we did not examine associations between *PAI-1* gene haplotypes and the risk of these diseases due to insufficient haplotype data. It remains unclear whether other *PAI-1* gene mutations can lead to changes in its expression. In terms of the genetic causes of disease, haplotypes can provide more critical information than the corresponding single SNP.

## 5 Conclusion

In conclusion, the present meta-analysis suggested that *PAI-1* 4G/5G (rs1799889) gene polymorphisms might be associated with the risk of MetS, but no evidence was detected in AD and female infertility. However, our results of this meta-analysis should be interpreted with caution owing to the small sample size and small number of studies available. Well-designed studies with large sample sizes in different ethnicities are warranted.

## Acknowledgments

We thank all the participants for their cooperation.

## Author contributions

**Conceptualization:** Bing Xu.

**Data curation:** Xin Zhang, Bing Xu.

**Formal analysis:** Xin Zhang, Bai Gao.

**Investigation:** Xin Zhang, Bai Gao.

**Methodology:** Xin Zhang, Bai Gao.

**Project administration:** Bing Xu.

**Resources:** Bai Gao.

**Software:** Xin Zhang.

**Supervision:** Bing Xu.

**Validation:** Bai Gao.

**Visualization:** Bai Gao.

**Writing – original draft:** Xin Zhang, Bing Xu.

**Writing – review & editing:** Bing Xu.
